# A Novel Inhibitor INF 39 Promotes Osteogenesis via Blocking the NLRP3/IL-1*β* Axis

**DOI:** 10.1155/2022/7250578

**Published:** 2022-07-13

**Authors:** Wenxiang Chen, Pan Tang, Shunwu Fan, Xuesheng Jiang

**Affiliations:** ^1^Department of Orthopaedics, Huzhou Central Hospital, Affiliated Central Hospital Huzhou University, Huzhou, Zhejiang, China; ^2^Department of Orthopaedics, Sir Run Run Shaw Hospital, School of Medicine, Zhejiang University, Hangzhou, Zhejiang, China

## Abstract

**Purpose:**

A balance between osteoblasts and osteoclasts is essential to maintain skeletal integrity, regulating bone metabolism and bone remodeling. The nucleotide binding oligomerization domain, leucine-rich repeat and pyrin domain containing protein 3 (NLRP3) inflammasome is known as a cytosolic complex involved in producing proinflammatory cytokines consisting of interleukin- (IL-) 1*β*, which accelerates the occurrence of osteoporosis. Therefore, we aimed to investigate the effect of a novel NLRP3 inhibitor INF 39 on bone formation and bone resorption. *Material and Methods*. Cell viability of INF 39-treated osteoclasts and calvarial osteoblasts was tested by CCK-8 assays. Quantitative RT-PCR (qRT-PCR) was used to evaluate gene expression level during osteoblast and osteoclast formation. Western blot analysis was used to determine the effect of INF 39 on osteogenic and osteoclast-related proteins.

**Result:**

It was shown that INF 39 promotes osteoblast differentiation via inhibiting NLRP3, thereby reducing the production of IL-1*β* dependent on NLRP3 *in vitro*. However, RANKL-induced osteoclast differentiation is not influenced by INF 39 *in vitro*.

**Conclusion:**

Our study suggests that NLRP3 could be a new target and INF 39 may be a potential option for prevention and treatment of osteoporosis.

## 1. Introduction

Tight interplay between osteoblasts and osteoclasts regulates bone metabolism and bone remodeling, which is the process of removing older bone and replacing it with a new one [1]. A balance between the two cells is essential to maintain skeletal integrity, and any disturbance in this balance will result in the generation of bone pathology, the most common being postmenopausal osteoporosis in aging women leading to increased fracture incidence by impairment of bone strength [2]. There are considerable issues with the use of currently available drugs for the treatment of osteoporosis. Antiresorptive drugs, such as diphosphonate, are very efficient at reducing osteoclast numbers and activity, resulting in markedly decreased bone resorption, while they also profoundly inhibit bone formation [3, 4]. Bone formation-acceleration drugs, such as teriparatide, can promote osteoblast proliferation and increase bone mass effectively, but they are coupled with increased bone resorption.

Studies have demonstrated that the proinflammatory cytokine interleukin- (IL-) 1*β* inhibits mineralization and osteogenesis [5, 6] and decreased IL-1*β* attenuates bone loss and represses osteoclast formation and activity [7–9]. The nucleotide binding oligomerization domain, leucine-rich repeat and pyrin domain containing protein 3 (NLRP3) inflammasome is known as a cytosolic complex involved in producing proinflammatory cytokines consisting of IL-1*β* and IL-18. Considerable models driving activation of the NLRP3 inflammasome have been proposed, including K^+^ efflux, ROS production, mitochondrial dysfunction, and lysosomal rupture [10]. The NLRP3 protein is assembled and activated in response to the process wherein Toll-like receptors (TLRs) recognize pathogen-associated molecular patterns (PAMPs) or damage-associated molecular patterns (DAMPs) to activate the nuclear factor-*κ*B (NF-*κ*B) signaling pathway. Then, procaspase-1 is recruited and autocleaves itself by releasing active caspase-1 to cleave pro-IL-1*β* and pro-IL-18 to promote interleukin maturation and subsequent release, which sets up the inflammation [11–13].

There is increasing evidence implicating that NLRP3 inflammasome is a potential target for therapy because of its involvement in several diseases, such as osteoporosis, osteoarthritis, and rheumatoid arthritis [14–17]. INF 39 is a novel acrylate compound inhibiting NLRP3 ATPase activity. van der Schot and Bonvin [18] found that INF 39 was a nontoxic, irreversible NLRP3 inhibitor exerting a role in decreasing IL-1*β* release from macrophage *in vitro*. Meanwhile, the *in vivo* study confirmed that the compound was able to alleviate the effect of dinitrobenzenesulfonic acid-induced colitis in rats. It is also corroborated that direct inhibition of NLRP3 inflammasome with INF 39 is effective for the treatment of bowel inflammation and has more advantages than caspase-1 inhibition or IL-1*β* receptor blockade [19]. However, the role of INF 39 in bone homeostasis is unclear. Here, we aimed to investigate the effect of INF 39 on bone homeostasis and explore the underlying molecular mechanism.

## 2. Materials and Methods

### 2.1. Reagents

Eagle's minimal essential medium with alpha modification (*α*-MEM), FBS, and penicillin/streptomycin were purchased from Gibco-BR (Carlsbad, CA, USA). INF 39 was purchased from Selleck (Shanghai, China), and DMSO was purchased from Sigma-Aldrich (St. Louis, MO, USA). CCK-8 (Cell Counting Kit-8) was obtained from Dojindo Molecular Technology (Kumamoto, Japan). Recombinant soluble mouse macrophage colony-stimulating factor (M-CSF) and mouse receptor activator of nuclear factor-*κ*B ligand (RANKL) were obtained from R&D Systems (Minneapolis, MN, USA). Specific antibodies against NLRP3, IL-1*β*, c-Fos, nuclear factor of activated T-cells c1 (NFATc1), GAPDH, *β*-actin, and *β*-tubulin were obtained from Cell Signaling Technology (Beverly, MA, USA), and antibodies against alkaline phosphatase (ALP) were purchased from Abcam (Cambridge, MA, USA). Tartrate-resistant acid phosphatase- (TRAP-) staining kit and all other reagents were purchased from Sigma-Aldrich, unless otherwise stated.

### 2.2. Mice

All experimental procedures were approved by the Ethics Review Committee for Animal Experimentation of Sir Run Run Shaw Hospital. The mice were from C57BL/6 background and maintained at the specific pathogen-free animal care facility and housed in a room at 24 ± 2°C, with 50 ± 5% humidity and a 12-hour light/dark cycle, with lights on from 7:00 a.m. to 7:00 p.m. All mice were allowed access to drinking water and regular rodent chow *ad libitum*.

### 2.3. Cell Culture, Osteoblast, and Osteoclast Differentiation

Primary calvarial osteoblasts were obtained as described previously [4, 20]. Calvariae were from 2- to 4-day-old C57BL/6 mice, which were digested overnight with collagenase type II in Dulbecco's modified Eagle's medium (DMEM) containing 10% FBS while being incubated at 37°C. After centrifugation, the cells were collected and seeded into culture dishes in DMEM containing 10% FBS. After 4 days, cells were reseeded in 12-well dishes and cultured in osteogenic medium (1 mM *β*-glycerophosphate and 5 *μ*M L-ascorbic acid 2-phosphate) with different concentrations of INF 39 (0, 25, 50, and 100 nM). After 7 days, ALP staining was performed, and the level of ALP in the cells and cell culture medium was determined according to the manufacturer's instructions. Following the treatment with osteogenic medium for 21 days using INF 39 at concentrations of 0, 25, 50, and 100 nM, cells were gently washed twice with PBS, fixed in 4% paraformaldehyde for 20 min, and stained with Alizarin Red S solution (ARS) (ScienCell, San Diego, CA, USA). Untreated cells were considered as a control. The images of extracellular matrix mineralization nodules were obtained using an inverted microscope with a digital camera. Quantification was performed according to the manufacturer's instructions (ScienCell, San Diego, CA, USA) [21].

Primary bone marrow-derived monocytes (BMMs) were cultured from male 6-week-old C57BL/6 mice as described previously [22]. BMMs were flushed out from the femoral and tibial bone marrow, then resuspended, plated to a 10 cm dish, and stimulated with an appropriate culture medium (*α*-MEM containing 10% FBS, 1% penicillin/streptomycin, 25 ng/mL M-CSF, and 50 ng/mL RANKL). BMMs were seeded in 96-well dishes at a density of 1 × 10^4^cells per well with 25 ng/mL M-CSF, 50 ng/mL RANKL, and different concentrations of INF 39 (0, 25, 50, and 100 nM). Osteoclasts were visualized by TRAP staining.

### 2.4. Cytotoxicity Assay

The cytotoxic effects of INF 39 on primary calvarial osteoblasts and BMMs were determined using the CCK-8 assay. BMMs were seeded in 96-well plates at a density of 2 × 10^4^cells per well in triplicate in the presence of 25 ng/mL M-CSF for 24 hours. Similarly, calvarial osteoblasts were seeded in 96-well plates at a density of 2 × 10^4^cells per well in triplicate with DMEM medium. Cells were then treated with different concentrations of INF 39 (0, 12.5, 25, 50, 100, and 200 nM) for 48 or 96 hours. Then, 10 *μ*L of CCK-8 buffer was added to each well, and the plates were incubated for an additional 2 hours. Afterward, the absorbance was measured at 450 nm wavelength (650 nm reference) on an ELX800 absorbance microplate reader (BioTek Instruments, Winooski, VT, USA).

### 2.5. RNA Extraction and Quantitative RT-PCR

Quantitative RT-PCR (qRT-PCR) was used to evaluate gene expression levels during osteoclast formation. Following the RANKL-induced osteoclastogenesis, mouse BMMs were treated with different doses of INF 39 (0, 25, 50, and 100 nM) for 5 days. Total RNA was isolated according to the manufacturer's protocol. Complementary DNA (cDNA) was synthesized using 1 *μ*g of RNA obtained from each sample, 2 *μ*L of 5x PrimeScript RT Master Mix (Takara Bio, Otsu, Japan), and 4 *μ*L of RNAse-free double-distilled water (ddH_2_O) in a total volume of 10 *μ*L. RT-PCR was performed using an ABI Prism 7500 system (Applied Biosystems, Foster City, CA, USA) with SYBR Green QPCR Master Mix (Takara Bio, Otsu, Japan). The total volume (10 *μ*L) of each PCR comprised 5 *μ*L SYBR Green QPCR Master Mix, 2 *μ*L double-distilled water (ddH_2_O), 2 *μ*L cDNA, and 1 *μ*L (10 *μ*M) each of forward and reverse primers. RT-PCR was performed at 95°C for 10 min (activation), followed by 45 cycles at 95°C for 10 s, 60°C for 20 s, and 72°C for 20 s (amplification) and a final extension at 72°C for 90 s. Specificity of amplification was verified by performing RT-PCR and analyzing the melting curves [23, 24]. Gene expression assay was normalized to GAPDH. Mouse *GAPDH*, *cathepsin K* (*CTSK*), *c-Fos*, *NFATc1*, *Runx2*, *ALP*, *COL1a*, *OCN*, and *NLRP3* primer sequences are presented in Supplemental Table S1.

### 2.6. Western Blot Analysis

To determine the effect of INF 39 on c-Fos and NFATc1, BMMs were treated with 25 ng/mL M-CSF and 50 ng/mL RANKL and with 25, 50, or 100 nM INF 39 or without it for 6 days. To determine the effect of INF 39 on Runx2 and ALP, primary calvarial osteoblasts were treated with 25, 50, or 100 nM INF 39 or without it, for 7 days. Total protein was extracted from the cultured cells using radioimmunoprecipitation assay (RIPA) lysis buffer (Sigma-Aldrich). Lysates were centrifuged at 12,000 g for 10 min, and the supernatants were collected. Proteins were separated by 10% SDS-PAGE and transferred to polyvinylidene difluoride (PVDF) membranes (Bio-Rad, Hercules, CA, USA). The membranes were blocked in 5% nonfat dry milk in TBST at room temperature for 1 hour and incubated with the primary antibodies overnight at 4°C. Protein bands were visualized using LAS-4000 Science Imaging System (Fujifilm, Tokyo, Japan), and the obtained images were analyzed with ImageJ software (NIH, Bethesda, MD, USA) [25, 26].

### 2.7. Small-Interfering RNA (siRNA) Transfection

Negative control (NC) siRNA and NLRP3 siRNA were purchased from GenePharma (Shanghai, China). siRNA was transfected into primary calvarial osteoblasts using Lipofectamine 3000 (Invitrogen, Carlsbad, CA, USA) following the manufacturer's instructions; then, osteoblast differentiation was performed.

### 2.8. ELISA

The whole blood was collected by orbital venous of the mice at the end of the treatment period. Serum was prepared and stored at -80°C until used. ELISA was used to measure serum level of IL-1*β* (mouse IL-1*β* ELISA kit; Lianke, Hangzhou, China), according to the manufacturer's instructions.

### 2.9. Statistical Analysis

Statistical analyses were performed using Prism 7 (GraphPad Software, Inc., San Diego, CA, USA). The results were expressed as mean ± SD. Statistical difference was assessed by Student's *t* test or one-way ANOVA. Values of *P* < 0.05 were regarded as statistically significant.

## 3. Results

### 3.1. INF 39 Positively Regulates Osteoblast Activity via Repressing NLRP3 In Vitro

**Figure 1:**
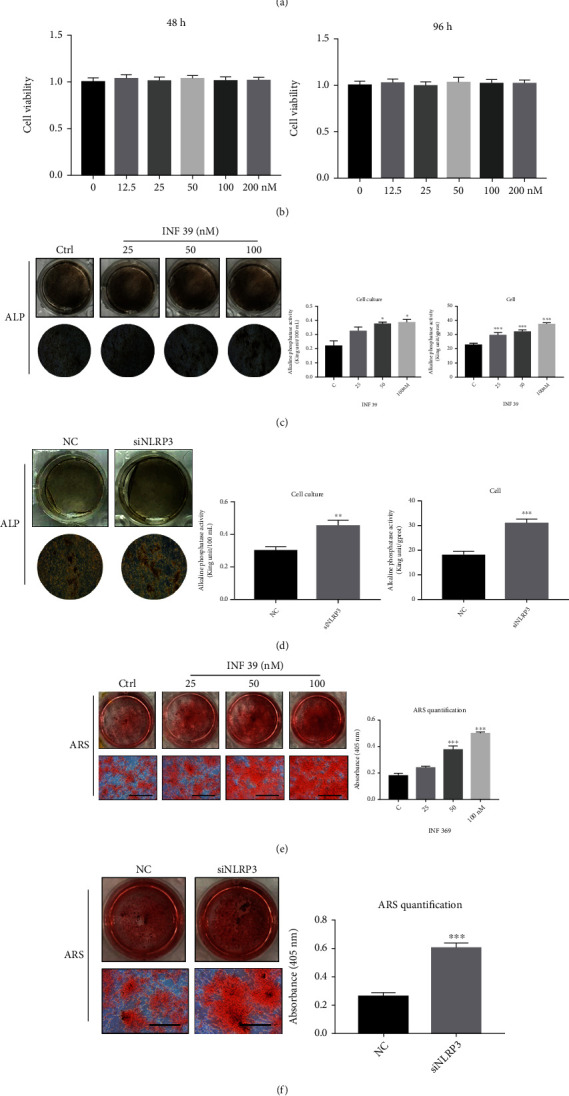
INF 39 increases osteoblast activity. (a) The structure of INF 39. (b) Cell viability of INF 39-treated mouse calvarial osteoblasts tested by CCK-8 assay at 48 and 96 hours. (c) The ALP activity of mouse calvarial osteoblasts, ALP staining, quantification in cell culture, and cell with various concentrations of INF 39. (d) ALP staining, quantification in cell culture, and cell after knockdown of NLRP3. (e) Alizarin Red S staining and OD values of 405 nm obtained for mineralized matrix solutions following the treatment with INF 39. (f) Alizarin Red S staining and OD values obtained for mineralized matrix solutions after knockdown of NLRP3. Data are shown as the mean ± SD. ^∗^*P* < 0.01, ^∗∗^*P* < 0.01, and ^∗∗∗^*P* < 0.005, compared with the controls.

To determine the potential role of INF 39 in osteoblasts, we assessed the proliferation and differentiation of primary calvarial osteoblasts harvested from neonatal mice. Cell viability of INF 39-treated calvarial osteoblasts was tested by CCK-8 assays at 48 and 96 hours. It was shown that no cytotoxicity was at the dose of 200 nM (Figure 1(b)). Next, we investigated the effect of INF 39 in osteoblast differentiation from preosteoblasts to mature osteoblasts. Calvarial osteoblasts treated with different concentrations of INF 39 (25, 50, and 100 nM) were exhibited to induce an increase of the ALP activity in the culture medium and cell (Figure 1(c)). In addition, we explored the effect of INF 39 on the deposition of minerals in the extracellular matrix by using Alizarin Red S staining. More calcified nodules were examined on day 21 following the treatment with 25, 50, and 100 nM INF 39, compared with those in the controls. Therefore, the mineralizing capacity was strengthened, which was consistent with the change of ALP activity in the prior experiment (Figure 1(e)). Furthermore, we compared the expression of ALP and the deposition of minerals in the extracellular matrix between controls and NLRP3 knockdown osteoblasts and found that both the ALP activity and the deposition of minerals in the extracellular matrix were significantly increased after NLRP3 knockdown (Figures 1(d) and 1(f)).

**Figure 2:**
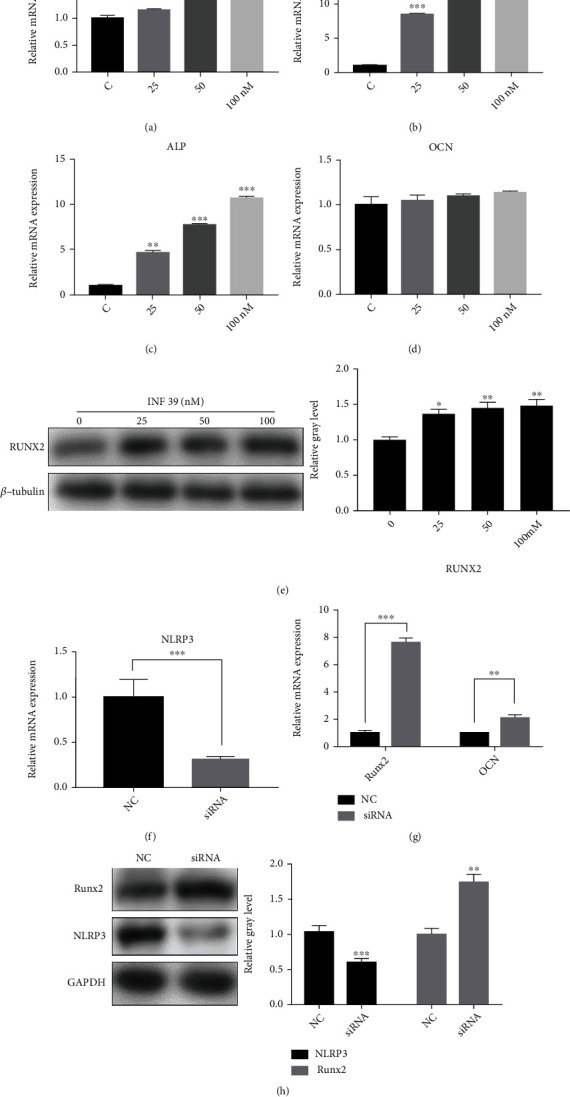
Repressing NLRP3 promotes osteoblast-related gene expression. (a–d) Expression of the osteoblast-specific genes *Runx2*, *COL1a*, *ALP*, and *OCN* in mouse calvarial osteoblasts treated with various concentrations of INF 39 (*n* = 3). Runx2: runt-related transcription factor 2; COL1a: collagen type 1*α*; ALP: alkaline phosphatase; OCN: osteocalcin. (e) The gray level of RUNX2 was quantified and normalized to *β*-tubulin using ImageJ (*n* = 3). (f) Expression of *NLRP3* mRNA after knockdown of INF 39 (*n* = 3). (g) Expression of the osteoblast-specific genes *Runx2* and *OCN* in mouse calvarial osteoblasts after knockdown of NLRP3 (*n* = 3). (h) The gray level of Runx2 and NLRP3 was quantified and normalized to GAPDH using ImageJ (*n* = 3). Data are shown as the mean ± SD. ^∗^*P* < 0.05, ^∗∗^*P* < 0.01, and ^∗∗∗^*P* < 0.005, compared with the controls.

To further evaluate the effect of INF 39 on specific gene expressions of mouse calvarial osteoblasts, we first examined the mRNA expression of osteoblast-specific genes *Runx2*, *COL1a*, *ALP*, and *OCN* by qRT-PCR analysis (Figures 2(a)–2(d)). It was shown that the expression of *Runx2*, *COL1a*, and *ALP* mRNA in osteoblasts was remarkably increased after treatment with 25, 50, and 100 nM INF 39 compared with that in the controls. However, the mRNA expression of *OCN* was not obviously changed after treatment with different INF 39 concentrations. Moreover, the expression of RUNX2 was upregulated with the increased concentrations of INF 39 (25, 50, and 100 nM) (Figure 2(e)). The results of the western blot analysis were consistent with those of the qRT-PCR analysis. Furthermore, we found that *Runx2* mRNA significantly increased and *OCN* mRNA was slightly elevated after knockdown of NLRP3 using siRNA against mouse osteoblast NLRP3 (Figures 2(f) and 2(g)). Moreover, the expression of Runx2 was also upregulated after knockdown of NLRP3 compared with that of the controls (Figure 2(h)). These results indicate that INF 39 upregulates osteoblast-related gene expression via inhibiting NLRP3 *in vitro*.

### 3.2. INF 39 Promotes Osteogenesis via Blocking the NLRP3/IL-1*β* Axis In Vitro

**Figure 3:**
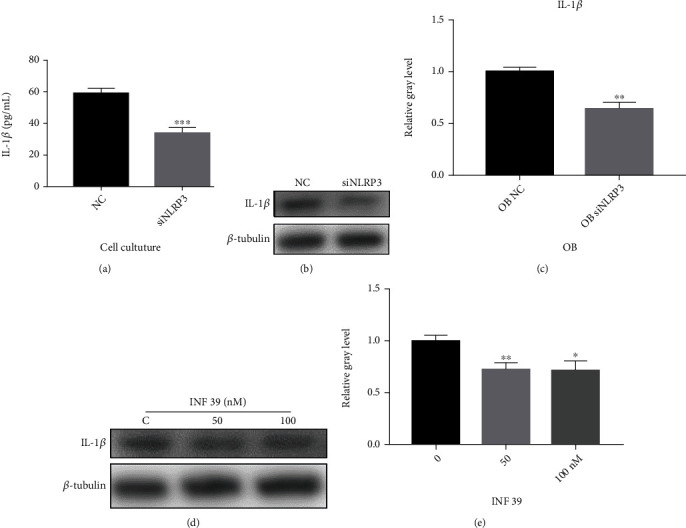
INF 39 promotes osteogenesis via inhibiting the NLRP3/IL-1*β* axis. (a) The concentration of IL-1*β* in the culture of osteoclasts, IL-1*β*: interleukin- (IL-) 1*β* (*n* = 3). (b, c) The gray level of IL-1*β* in NLRP3 knockdown osteoblasts for 7 d was quantified and normalized to *β*-tubulin using ImageJ (*n* = 3). (d, e) The gray level of IL-1*β* in osteoblasts treated with various concentrations of INF 39 for 7 d was quantified and normalized to *β*-tubulin using ImageJ (*n* = 3). ^∗^*P* < 0.05, ^∗∗^*P* < 0.01, and ^∗∗∗^*P* < 0.005.

To further clarify the underlying mechanisms, we tested the concentration of IL-1*β* in the osteoblast medium. We detected that the concentration of IL-1*β* was remarkably reduced in the NLRP3 knockdown group compared with the negative control (NC) group (Figure 3(a)). Afterward, we found that the expression of IL-1*β* was downregulated in NLRP3 knockdown osteoblasts compared with the NC osteoblasts (Figures 3(b) and 3(c)). Similarly, the level of IL-1*β* in osteoblasts was significantly decreased with various concentrations of INF 39 (0, 50, and 100 nM) for 7 d (Figures 3(d) and 3(e)). These results suggest that osteogenesis is increased via blocking the NLRP3/IL-1*β* axis *in vitro*.

### 3.3. INF 39 Have No Notable Effect on Osteoclastogenesis In Vitro

**Figure 4:**
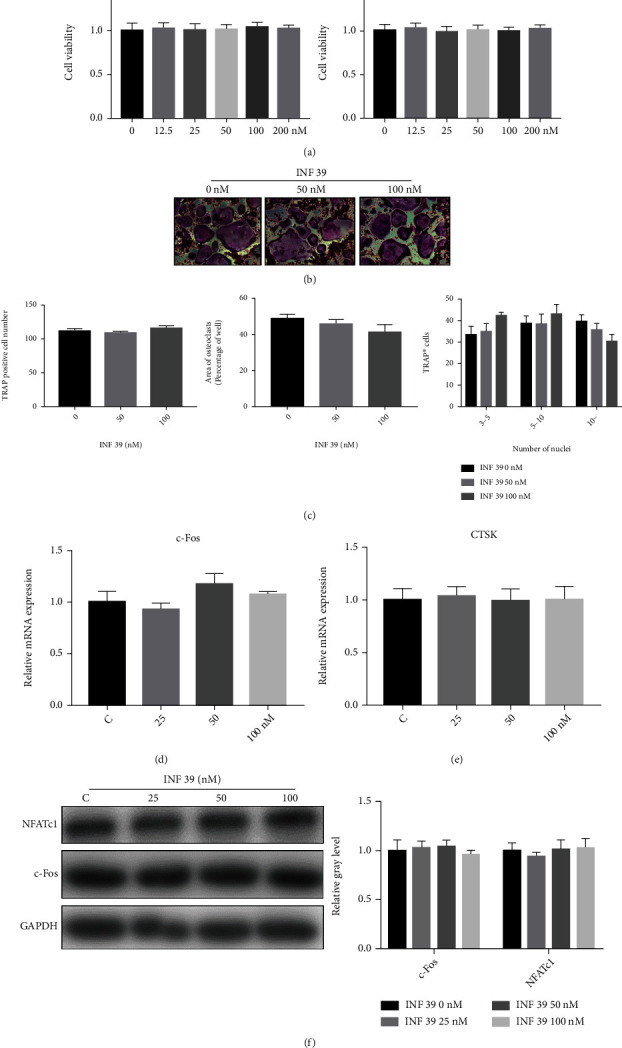
INF 39 does not have a remarkable effect on osteoclastogenesis. (a) Cell viability of INF 39-treated BMMs tested by CCK-8 assays at 48 and 96 hours. (b) TRAP staining of BMMs treated with various concentrations of INF 39, M-CSF (25 ng/mL), and RANKL (50 ng/mL) for 6 days. (c) Quantification of TRAP-positive multinuclear cells, osteoclast number, area of osteoclasts, and osteoclast size. (d, e) Expression of the osteoclast-specific genes *c-Fos* and *CTSK* in BMMs treated with various concentrations of INF 39 (*n* = 3). CTSK: cathepsin K. (f) The gray level of NFATc1 and c-Fos was quantified and normalized to GAPDH using ImageJ (*n* = 3).

We first assessed cell viability of INF 39-treated BMMs using CCK-8 assays at 48 and 96 hours, and no cytotoxicity was shown at the dose of 200 nM (Figure 4(a)). Next, we evaluated whether INF 39 affected osteoclast differentiation which derived from BMMs in the presence of M-CSF and RANKL. Then, we observed that osteoclast number, area of osteoclasts, and osteoclast size determined by TRAP stain were not significantly changed with different concentrations of INF 39 (0, 50, and 100 nM) (Figures 4(b) and 4(c)). Additionally, we examined the mRNA expression of *c-Fos* and *CTSK* in BMMs after being cultured for 4 days, and the mRNA expression did not vary after treatment with INF 39 (0, 25, 50, and 100 nM) (Figures 4(d) and 4(e)). Meanwhile, the expression of NFATc1 and c-Fos was not obviously different after being analyzed by using western blot (Figure 4(f)). These results suggest that RANKL-induced osteoclast differentiation is not influenced by INF 39 *in vitro*.

## 4. Discussions

**Figure 5:**
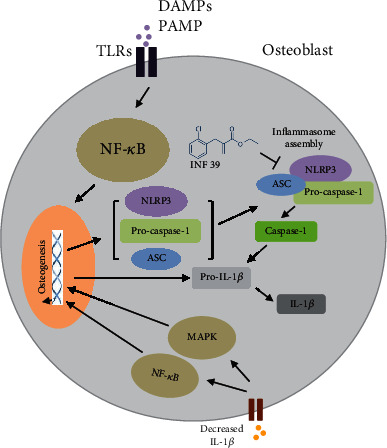
Schematic representation of experiments. INF 39 accelerates osteogenesis via blocking the NLRP3/IL-1*β* axis.

As the osteoporotic fracture brings a heavy burden on the family and society, prevention and therapy of osteoporosis become essential specifically for the aging population. At present, therapeutic methods for osteoporosis are limited, and the effect of the available drugs is not that desirable. Therefore, we focus on the research of the novel compound, INF 39, which potentially targets osteoporosis. We investigated the effect of INF 39 on bone formation through performing a series of experiments *in vitro*. Our results showed that INF 39 promoted osteoblast differentiation via inhibiting NLRP3, thereby reducing the production of IL-1*β* dependent on NLRP3 *in vitro* (Figure 5).

At the beginning, we performed ALP and ARS staining [27, 28] to determine that INF 39 accelerated osteogenesis in both the early and late stages in a dose-dependent manner. The osteoblast-related genes, including *ALP*, *COL1a*, *Runx2*, and *OCN* [29, 30], had a higher expression in the INF 39-treated groups than in the control group. Accordingly, the expression of specific proteins, such as ALP and Runx2, was elevated in the INF 39-treated groups than in the control group. All these observations collectively suggested that INF 39 had a positive effect on osteoblast formation. Subsequently, we investigated the underlying mechanism of the effect of INF 39 on osteogenesis. Primary calvarial osteoblasts treated with NLRP3 siRNA showed that the ALP expression and calcium deposits were significantly increased compared with those in the normal control group. Moreover, the key transcription factor of osteoblast formation Runx2 also exhibited an apparent rise in the NLPR3 knockdown group in contrast to the normal control group.

The NLRP3 inflammasome is known as a major producer of cleaved IL-1 family cytokines, and activation of NLRP3 induces NLRP3-dependent IL-1*β* secretion, leading to inflammatory disease via the NF-*κ*B pathway, leading to inflammatory disease via NF-*κ*B and p38/MAPK pathways [31–35]. Interleukin-1*β*, a prototypic inflammatory cytokine, accelerates acute or chronic inflammation, resulting in the initiation and progress of osteopenia and osteoporosis [36]. Therefore, the expression of IL-1*β* in the osteoblasts was examined and was shown to decline with NLRP3 knockdown. To verify whether osteoblast formation was influenced by the variation of the expression of IL-1*β*, we tested the concentrations of IL-1*β* in the osteoblast medium, and it was shown that the level of IL-1*β* was lower in the osteoblast NLRP3 knockdown group than in the normal group.

Our study first investigated the effect of the novel compound INF 39 on bone metabolism and found that INF 39 plays an important role in increasing bone formation. Thus, this novel compound may have more potential advantages than other drugs already on the market. However, some limitations could not be avoided in our study. For instance, we have not investigated the effect of INF 39 on skeletal homeostasis *in vivo*. Other mechanisms of INF 39 regulating osteogenesis and osteoclastogenesis remain to be further explored in the future.

## 5. Conclusions

Our study reveals that INF 39 promotes osteoblast formation via blocking NLRP3 leading to downregulation of the expression of IL-1*β in vitro*. Our study suggests that NLRP3 could be a new target and INF 39 may be a potential option for prevention and treatment of osteoporosis.

## Data Availability

The data used to support the findings of this study are included within the article.

## Supplementary Materials

Supplementary Materials Supplementary Table 1: primers used in the real-time PCR.

## References

[1] Fukuda M., Yoshizawa T., Karim M. (2018). SIRT7 has a critical role in bone formation by regulating lysine acylation of SP7/osterix. *Nature Communications*.

[2] Luther J., Yorgan T. A., Rolvien T. (2018). Wnt1 is an Lrp5-independent bone-anabolic Wnt ligand. *Science Translational Medicine*.

[3] Lotinun S., Kiviranta R., Matsubara T. (2013). Osteoclast-specific cathepsin K deletion stimulates S1P-dependent bone formation. *The Journal of Clinical Investigation*.

[4] Chen W., Xie Z., Tang P. (2019). The emerging role of IMD 0354 on bone homeostasis by suppressing osteoclastogenesis and bone resorption, but without affecting bone formation. *Cell Death & Disease*.

[5] Jeschke A., Catala-Lehnen P., Sieber S. (2015). Sharpin controls osteogenic differentiation of mesenchymal bone marrow cells. *Journal of Immunology (Baltimore, Md. : 1950)*.

[6] Liu C., Xiong H., Chen K., Huang Y., Huang Y., Yin X. (2016). Long-term exposure to pro-inflammatory cytokines inhibits the osteogenic/dentinogenic differentiation of stem cells from the apical papilla. *International Endodontic Journal*.

[7] Vijayan V., Khandelwal M., Manglani K., Singh R. R., Gupta S., Surolia A. (2013). Homocysteine alters the osteoprotegerin/RANKL system in the osteoblast to promote bone loss: pivotal role of the redox regulator forkhead O1. *Free Radical Biology & Medicine*.

[8] Guan H., Zhao L., Cao H., Chen A., Xiao J. (2015). Epoxyeicosanoids suppress osteoclastogenesis and prevent ovariectomy-induced bone loss. *FASEB Journal : Official Publication of the Federation of American Societies for Experimental Biology*.

[9] Zhao L., Cai C., Wang J. (2017). Dihydromyricetin protects against bone loss in ovariectomized mice by suppressing osteoclast activity. *Frontiers in Pharmacology*.

[10] He Y., Hara H., Núñez G. (2016). Mechanism and regulation of NLRP3 inflammasome activation. *Trends in Biochemical Sciences*.

[11] Jo E. K., Kim J. K., Shin D. M., Sasakawa C. (2016). Molecular mechanisms regulating NLRP3 inflammasome activation. *Cellular & Molecular Immunology*.

[12] Chen J., Chen Z. J. (2018). PtdIns4P on dispersed trans-Golgi network mediates NLRP3 inflammasome activation. *Nature*.

[13] Suetomi T., Willeford A., Brand C. S. (2018). Inflammation and NLRP3 inflammasome activation initiated in response to pressure overload by Ca^2+^/calmodulin-dependent protein kinase II *δ* signaling in cardiomyocytes are essential for adverse cardiac remodeling. *Circulation*.

[14] Jin C., Frayssinet P., Pelker R. (2011). NLRP3 inflammasome plays a critical role in the pathogenesis of hydroxyapatite-associated arthropathy. *Proceedings of the National Academy of Sciences of the United States of America*.

[15] An Y., Zhang H., Wang C. (2019). Activation of ROS/MAPKs/NF-*κ*B/NLRP3 and inhibition of efferocytosis in osteoclast-mediated diabetic osteoporosis. *The FASEB Journal*.

[16] Snouwaert J. N., Nguyen M., Repenning P. W. (2016). An NLRP3 mutation causes arthropathy and osteoporosis in humanized mice. *Cell Reports*.

[17] Xu L., Shen L., Yu X., Li P., Wang Q., Li C. (2020). Effects of irisin on osteoblast apoptosis and osteoporosis in postmenopausal osteoporosis rats through upregulating Nrf2 and inhibiting NLRP3 inflammasome. *Experimental and Therapeutic Medicine*.

[18] van der Schot G., Bonvin A. M. (2015). Performance of the WeNMR CS-Rosetta3 web server in CASD-NMR. *Journal of Biomolecular NMR*.

[19] Pellegrini C., Fornai M., Colucci R. (2018). A comparative study on the efficacy of NLRP3 inflammasome signaling inhibitors in a pre-clinical model of bowel inflammation. *Frontiers in Pharmacology*.

[20] Yagishita N., Yamamoto Y., Yoshizawa T. (2001). Aberrant growth plate development in VDR/RXR gamma double null mutant mice. *Endocrinology*.

[21] Choi J. Y., Lai J. K., Xiong Z. M. (2018). Diminished canonical *β*-catenin signaling during osteoblast differentiation contributes to osteopenia in progeria. *Journal of Bone and Mineral Research: the Official Journal of the American Society for Bone and Mineral Research*.

[22] Chen S., Jin G., Huang K. M. (2015). Lycorine suppresses RANKL-induced osteoclastogenesis in vitro and prevents ovariectomy-induced osteoporosis and titanium particle-induced osteolysis in vivo. *Scientific Reports*.

[23] Chen X., Zhi X., Pan P. (2017). Matrine prevents bone loss in ovariectomized mice by inhibiting RANKL-induced osteoclastogenesis. *FASEB Journal : Official Publication of the Federation of American Societies for Experimental Biology*.

[24] Bouxsein M. L., Boyd S. K., Christiansen B. A., Guldberg R. E., Jepsen K. J., Müller R. (2010). Guidelines for assessment of bone microstructure in rodents using micro-computed tomography. *Journal of Bone and Mineral Research*.

[25] Jie Z., Shen S., Zhao X. (2019). Activating *β*‐catenin/Pax6 axis negatively regulates osteoclastogenesis by selectively inhibiting phosphorylation of p38/MAPK. *FASEB Journal : Official Publication of the Federation of American Societies for Experimental Biology*.

[26] Dempster D. W., Compston J. E., Drezner M. K. (2013). Standardized nomenclature, symbols, and units for bone histomorphometry: a 2012 update of the report of the ASBMR Histomorphometry Nomenclature Committee. *Journal of Bone and Mineral Research: the Official Journal of the American Society for Bone and Mineral Research*.

[27] Greenblatt M. B., Park K. H., Oh H. (2015). CHMP5 controls bone turnover rates by dampening NF-*κ*B activity in osteoclasts. *The Journal of Experimental Medicine*.

[28] Xie Z., Yu H., Sun X. (2018). A novel diterpenoid suppresses osteoclastogenesis and promotes osteogenesis by inhibiting Ifrd1-mediated and I*κ*B*α*-mediated p65 nuclear translocation. *Journal of Bone and Mineral Research*.

[29] Guo Y. C., Wang M. Y., Zhang S. W. (2018). Ubiquitin-specific protease USP34 controls osteogenic differentiation and bone formation by regulating BMP2 signaling. *The EMBO Journal*.

[30] Pal S., Porwal K., Khanna K. (2019). Oral dosing of pentoxifylline, a pan-phosphodiesterase inhibitor restores bone mass and quality in osteopenic rabbits by an osteogenic mechanism: a comparative study with human parathyroid hormone. *Bone*.

[31] Weichand B., Popp R., Dziumbla S. (2017). S1PR1 on tumor-associated macrophages promotes lymphangiogenesis and metastasis via NLRP3/IL-1*β*. *The Journal of Experimental Medicine*.

[32] Barry R., John S. W., Liccardi G. (2018). SUMO-mediated regulation of NLRP3 modulates inflammasome activity. *Nature Communications*.

[33] Grebe A., Hoss F., Latz E. (2018). NLRP3 inflammasome and the IL-1 pathway in atherosclerosis. *Circulation Research*.

[34] Kasper L., König A., Koenig P. A. (2018). The fungal peptide toxin Candidalysin activates the NLRP3 inflammasome and causes cytolysis in mononuclear phagocytes. *Nature Communications*.

[35] Marchetti C., Swartzwelter B., Gamboni F. (2018). OLT1177, a *β*-sulfonyl nitrile compound, safe in humans, inhibits the NLRP3 inflammasome and reverses the metabolic cost of inflammation. *Proceedings of the National Academy of Sciences of the United States of America*.

[36] Liu X., Zhu S., Cui J. (2014). Strontium ranelate inhibits titanium-particle-induced osteolysis by restraining inflammatory osteoclastogenesis in vivo. *Acta Biomaterialia*.

