# Supplementary material for: A Novel Inhibitor INF 39 Promotes Osteogenesis via Blocking the NLRP3/IL-1β Axis
Source: Biomed Res Int. 2022 Jul 13;2022:7250578. doi: 10.1155/2022/7250578 (PMC9300331; doi:10.1155/2022/7250578)
Supplement: Supplementary Materials — Supplementary Table 1: primers used in the real-time PCR. [file 7250578.f1.docx]

**Supplementary Table 1: Primers used in the real-time PCR**

| **Genes** | **Upstream (5′-3′)** | **Downstream (5′-3′)** |
| --- | --- | --- |
| ***GAPDH*** | ACCCAGAAGACTGTGGATGG | CACATTGGGGGTAGGAACAC |
| ***CTSK*** | CTTCCAATACGTGCAGCAGA | TCTTCAGGGCTTTCTCGTTC |
| ***c-Fos*** | CCAGTCAAGAGCATCAGCAA | AAGTAGTGCAGCCCGGAGTA |
| ***NFATc1*** | CCGTTGCTTCCAAAAATAACA | TGTGGGATGTGAACTCGGAA |
| ***ALP*** | CCAACTCTTTTGTGCCAGAGA | GGCTACATTGGTGTTGAGCTTTT |
| ***COL1a*** | CCCAGAGTGGAACAGCGATT | ATGAGTTCTTCGCTGGGGTG |
| ***OCN*** | GAGGGCAATAAGGTAGTGA ACAGA | AAGCCATACTGGTTTGATAGCTCG |
| ***Runx2*** | TTCTCCAACCCACGAATGCAC | CAGGTACGTGTGGTAGTGAGT |
| ***C/EBPα*** | ATGGTTTCGGGTCGCTGGAT | CTGACTCCCTCATCTTAGACGCAC |
| ***FHBP4*** | ATGAAATCACCGCAGACGA | ACACATTCCACCACCAGCTT |
| ***PPARγ*** | ACTCGCATTCCTTTGACAT | CGCACTTTGGTATTCTTGGAG |
| ***NLRP3*** | ATTACCCGCCCGAGAAAGG | TCGCAGCAAAGATCCACACAG |
